# Osteoid Osteoma of the Proximal Fibula: An Uncommon Location with the Indication for Open Surgery

**DOI:** 10.1155/2011/324650

**Published:** 2011-12-22

**Authors:** Bastian Marquass, Pierre Hepp, Jan Dirk Theopold, Nikolaus von Dercks, Thomas R. Blattert, Christoph Josten

**Affiliations:** ^1^Department of Trauma, Reconstructive and Plastic Surgery, University Hospital Leipzig, Liebigstraße 20, 04103 Leipzig, Germany; ^2^Department of Spine Surgery and Traumatology, Schwarzach Orthopaedic Clinic, Dekan-Graf-Straße 2-6, 94374 Schwarzach, Germany

## Abstract

*Purpose*. This is a case report of a patient with an osteoid osteoma of the proximal fibula. The objective is to illustrate a rare tumor location that requires open surgery due to closeness of neurological structures. *Methods*. Clinical and roentgenographic findings, treatment, and histological appearance are presented. *Results*. Local pain and swelling of the proximal fibula with improvement under salicylates led to the diagnosis of an osteoid osteoma, what was confirmed with an MRI scan. Due to proximity to the common peroneal nerve, we decided for open surgery. During the operation, the nerve was seen to cross the tumor site making it necessary to retract it to expose the entire tumor. Histologically, typical features of osteoid osteoma with a rather well-defined nidus surrounded by sclerotic bone were seen. A complete removal was performed. *Conclusion*. Osteoid osteomas of the proximal fibula are rare. When planning surgery, the common peroneal nerve must be identified, and its further distal course should be taken into account to avoid iatrogenic damage to the nerve.

## 1. Introduction

Osteoid osteoma is a well-described small benign osteoblastic tumor that primarily affects the diaphysis and metaphysis of the femur and tibia [1]. Other locations are the long bones of the upper extremity, the spine, and other rare locations, including the fibula. Osteoid osteoma affects men more than women (about 3 : 1), and 90% of the patients are younger than 25 years [2, 3].

It is clinically characterized by pain which is not related to physical exercise and is often exacerbated at night. Reduction of pain with nonsteroidal inflammatory drugs is not only a symptomatic treatment but also a diagnostic tool. The major differential diagnoses include Brodie abscess and, occasionally, stress fractures. Nidus osteoblasts stain well for COX-2 in histochemical analysis [4], which has been clinically proven based on the comparable effects of acetylsalicylic acid (inhibition of COX-1 and COX-2) and a selective COX-2 inhibitor [4, 5]. COX-2 is a key enzyme in the production of prostaglandins, particularly prostaglandin E2, which seems to be a major factor in pain induction for patients with osteoid osteoma [6]. Prostaglandin production in osteoid osteoma leads to an intense and chronic local inflammatory response with an accompanying periosteal reaction and sclerosis, seen in radiographs as a radiolucent nidus surrounded by reactive sclerosis. It is also described as the “double-density sign.”

As conservative therapy remains rare [7], the most common treatments involve en bloc resection, curettage, or minimally invasive techniques. Recent literature often presents less invasive techniques such as percutaneous CT-guided core drilling, radiofrequency, or laser thermoablation with good clinical results for treatment of osteoid osteoma [8–10].

We present a case of a 14-year-old girl, whose local pain and swelling of the proximal fibula with improvement under salicylates led to the diagnosis of an osteoid osteoma.

## 2. Case Report

**Figure 1:**
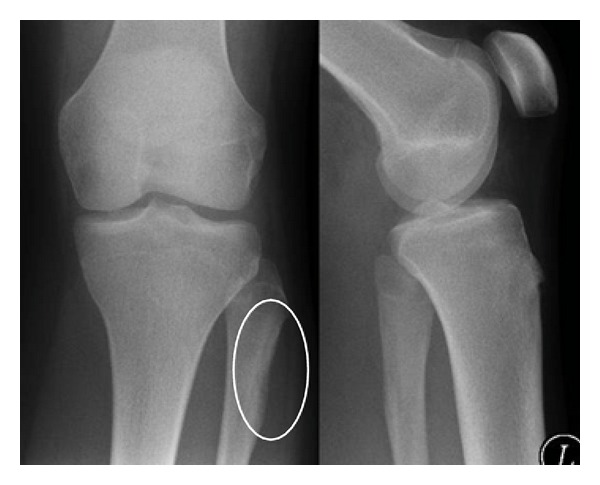
Preoperative X-ray showing local lucent nidus at the left proximal fibula.

**Figure 2:**
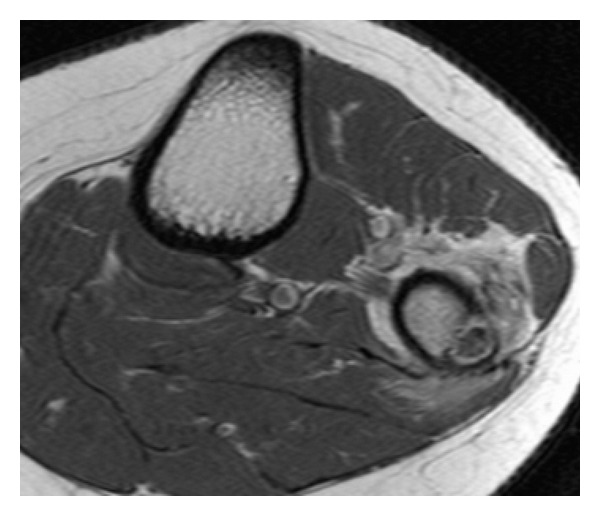
T1-weighted MRI scan showing the nidus surrounded by hyperintense tissue.

**Figure 3:**
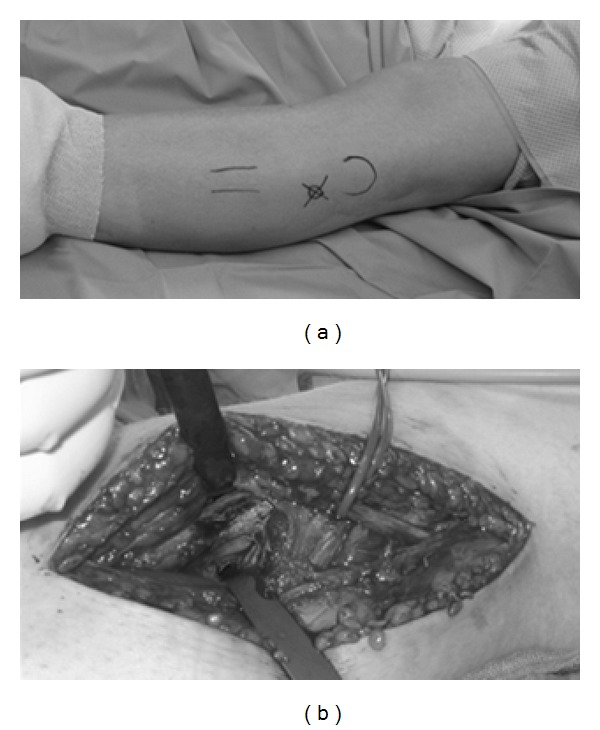
(a) The patient marked the most painful side distal to the fibular head. (b) The common peroneal nerve is in anatomical proximity to the tumor location.

**Figure 4:**
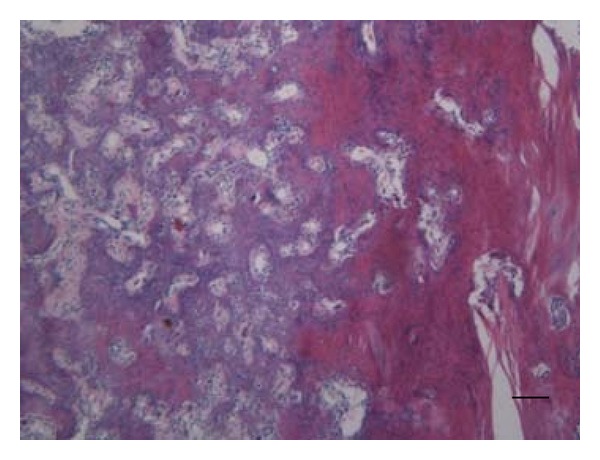
Histologically, a nidus of osteoid osteoma (left side) is seen bordered by sclerotic bone (right side). Bar: 100 *μ*m.

**Figure 5:**
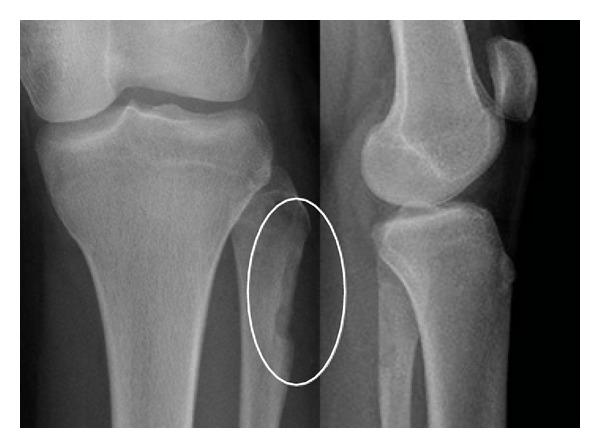
Postoperative radiographs demonstrate the resection zone of one-third of the shaft's width.

The patient's first symptoms were local swelling of the proximal lateral fibula and pain, mostly at night. In the first radiographs, a local lucent nidus at the left proximal lateral fibula was seen (Figure 1). Treatment with acetylsalicylic acid relieved the pain. After discontinuation of the medication, the symptoms recurred. The diagnosis of osteoid osteoma was confirmed with an MRI scan (Figure 2). The location was in the proximal part of the fibula on the lateral side in close topographic proximity to the common peroneal nerve, which winds around the fibular neck at that location. Therefore, the decision to perform open surgery was made. The patient marked her skin at the point of maximum pain (Figure 3(a)), and the incision was made at this location. After identifying the proximal portion of the common peroneal nerve at the posterior border of the short head of the biceps femoris, the nerve was retracted and dissection was performed along the course of the nerve in a distal direction (Figure 3(b)). An incision of the crural fascia was made, and the peroneal muscles were retracted anteriorly and the soleus muscle posteriorly. The tumor site was found just below the nerve and exactly at the preoperatively marked location. The tumor tissue, including the nidus of 0.7 cm, was removed by curettage. Histologically, the lesion showed the typical features of osteoid osteoma with a rather well-defined nidus surrounded by sclerotic bone. Bone trabeculae were surrounded by numerous activated osteoblasts without cytologic atypia (Figure 4). No postoperative neurological deficits were present. The patient was mobilized under full weight bearing. Radiographs showed complete removal of the osteoid osteoma, including the nidus, with a resulting lesion the size of one-third of the shaft width (Figure 5).

## 3. Discussion

Typical locations of osteoid osteomas are the long bones of the extremities, with the proximal femur being the most common site. However, in large studies, osteoid osteomas in the proximal fibula are rare. Campanacci et al. [11] found an incidence of 3% in a collective of 100 patients, while Sluga et al. [12] reports two cases in 126 patients (1.6%) as did Gangi et al. [9] in 114 patients (1.8%), and Cichon et al. found no osteoid osteoma of the fibula in a series of 74 patients [8]. We report about a patient with typical clinical and radiological symptoms in the proximal fibula, leading to a short history and early decision making. Because of the anatomical proximity to the common peroneal nerve and a 2.7 cm long tumor, we decided for open surgery.

Recent literature reports various techniques, including minimally invasive therapy. Thermocoagulation uses CT-controlled drilling of the nidus followed by radiofrequency thermoablation of tissue [13]. Comparable to this, Gangi et al. [14] attained good midterm results using interstitial laser ablation of the tissue. Another CT-guided procedure uses a drilling technique after guided targeting of the nidus [15, 16]. Assoun et al. [17] combine this procedure with ethanol injection in order to sclerose the remaining nidus. The drilling technique is also used in pediatric patients with good results [10]. However, when using a minimally invasive technique, care must be taken because large instruments may incur risk of neurologic and vascular injury, and the small needle core size increases the risk of incomplete nidus removal. Multiple passes might be required to complete the resection of the nidus; therefore, open surgery is recommended at a nidus size of more than one cm and a safety margin of less than one cm to neurogenic structures [18]. Furthermore, comparable results in outcome between the CT-guided technique and open surgery were demonstrated by Rosenthal et al. [19].

The “classic” surgical approach involves curettage or en bloc resection. In a comparison of both methods, Pfeiffer et al. [18] found a rate of recurrence of 0% for the en bloc technique and of 7% for the curettage, but found higher postoperative pain in more invasive techniques leading to a lower patient satisfaction. Therefore, the authors recommend curettage as a primary treatment and en bloc resection in case of recurrence.

In the presented case, we chose a surgical approach due to proximity to the common peroneal nerve. That was confirmed after exposing and identifying the nerve at the short head of the biceps femoris. The nerve was seen to cross the tumor site, and it was necessary to retract the nerve to expose the entire tumor. This made it possible to avoid iatrogenic damage to the nerve. Postoperatively, no neurological deficits were observed. The patient could be mobilized with full weight bearing.

In conclusion, osteoid osteoma has a predilection for tibia and femur but may be located in any bone. The assumed proximity to neural structures is an indication for open surgery. At the proximal fibula, the common peroneal nerve must be identified, and its further distal course should be taken into account when planning surgery.
